# Small extracellular vesicles secreted from senescent cells promote cancer cell proliferation through EphA2

**DOI:** 10.1038/ncomms15728

**Published:** 2017-06-06

**Authors:** Masaki Takasugi, Ryo Okada, Akiko Takahashi, David Virya Chen, Sugiko Watanabe, Eiji Hara

**Affiliations:** 1 Department of Molecular Microbiology, Research Institute for Microbial Diseases, Osaka University, Suita, Osaka 565-0871, Japan; 2 Division of Cancer Biology, Cancer Institute, Japanese Foundation for Cancer Research, Koto-ku, Tokyo 135-8550, Japan

## Abstract

Cellular senescence prevents the proliferation of cells at risk for neoplastic transformation. However, the altered secretome of senescent cells can promote the growth of the surrounding cancer cells. Although extracellular vesicles (EVs) have emerged as new players in intercellular communication, their role in the function of senescent cell secretome has been largely unexplored. Here, we show that exosome-like small EVs (sEVs) are important mediators of the pro-tumorigenic function of senescent cells. sEV-associated EphA2 secreted from senescent cells binds to ephrin-A1, that is, highly expressed in several types of cancer cells and promotes cell proliferation through EphA2/ephrin-A1 reverse signalling. sEV sorting of EphA2 is increased in senescent cells because of its enhanced phosphorylation resulting from oxidative inactivation of PTP1B phosphatase. Our results demonstrate a novel mechanism of reactive oxygen species (ROS)-regulated cargo sorting into sEVs, which is critical for the potentially deleterious growth-promoting effect of the senescent cell secretome.

## Abstract (web summary)

Although senescent cell secretome can promote the growth of surrounding cancer cells, the role of extracellular vesicles in this process has not been well understood. Here the authors show that ROS increase the sorting of EphA2 into extracellular vesicles in senescent cells, which promotes proliferation of cancer cells.

Cellular senescence is a state of irreversible cell cycle arrest that can be induced by a variety of potentially oncogenic stimuli and has therefore long been considered to suppress tumorigenesis in higher eukaryotes^1^,^2^,^3^. However, unlike apoptotic cells, senescent cells do not die immediately and therefore accumulate throughout the body during the ageing process^4^,^5^. Importantly, it has recently become apparent that senescent cells are not just cell cycle-arrested cells but also secrete various proteins such as, inflammatory cytokines, chemokines and matrix metalloproteinases into the extracellular space^6^,^7^,^8^. This newly recognized senescent phenotype, termed the senescence-associated secretory phenotype (SASP)^6^, occurs *in vivo* and has various biological effects that influence organismal homoeostasis^9^,^10^,^11^,^12^,^13^,^14^,^15^. Thus, a deeper understanding of the pathological and physiological roles of the SASP is crucial for clarifying the mechanisms underlying ageing and age-associated diseases, such as cancer.

In addition to secretory proteins, some senescent cells are reported to show increased secretion of small extracellular vesicles (sEVs)^16^. sEVs are heterogeneous populations of membrane vesicles^17^,^18^,^19^,^20^,^21^, including exosomes. Exosomes originate as the intra-luminal vesicles in late endosomal compartments by the inward budding of the endosomal membranes and are released from the cells upon fusion of the outer membrane with the plasma membrane. Emerging evidence indicates that sEVs play important roles in intercellular communication by serving as vehicles for the transfer of various cellular constituents (for example, proteins and nucleic acids) between cells^22^. In particular, some sEV proteins secreted from cancer cells have been shown to promote tumour development^22^,^23^,^24^,^25^,^26^,^27^.

Here we report that senescence not only increases the secretion of sEVs, but also alters their quality. We found that sEVs secreted from senescent cells have markedly altered protein composition, and exert pro-proliferative function on some cancer cell lines. This function was attributed at least partially to an enrichment of EphA2 in sEVs of senescent cells. sEV sorting of EphA2 is increased in senescent cells because of its enhanced phosphorylation resulting from oxidative inactivation of PTP1B phosphatase. Our findings revealed a novel mechanism of cell context-dependent cargo sorting into sEVs, which is important for the potentially deleterious pro-proliferative property of senescent cells.

## Results

### sEVs secreted from senescent cells are pro-proliferative

**Figure 1:**
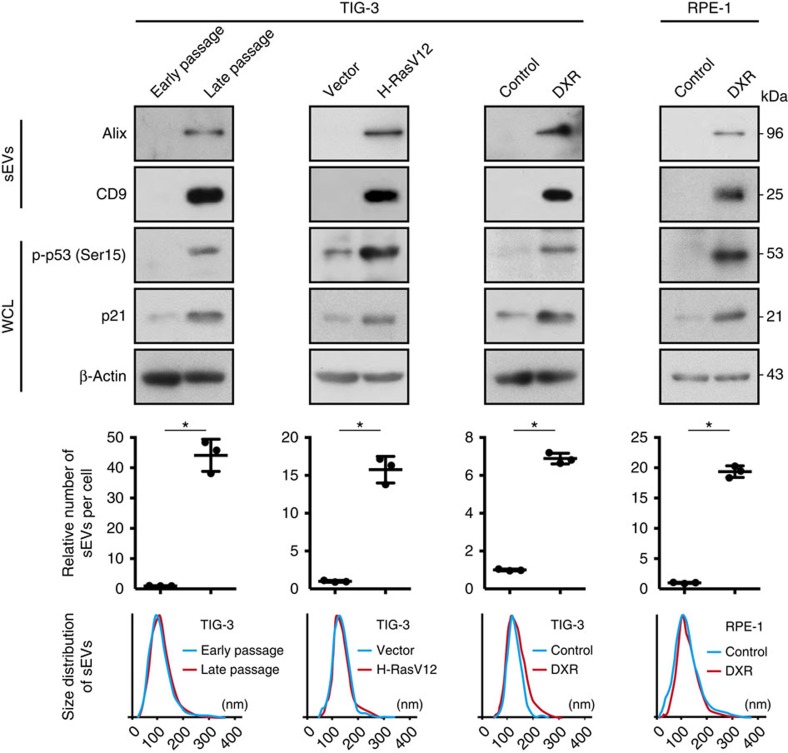
Senescence increases the secretion of sEVs. Immunoblotting of Alix (exosome marker) and CD9 (sEV marker) in the sEV fraction and of phospho-p53 (Ser15), p21 and β-actin in the whole cell lysates (WCL) of pre-senescent control and senescent cells. Senescence was induced by serial passage, oncogenic Ras expression or DXR-treatment in TIG-3 or RPE-1 cells. Dot plots show the relative numbers of sEVs per cell. The number of sEVs in the sEV fraction was quantified using NanoSight. The bottom graphs show the size distributions of sEVs secreted from control and senescent cells. Cell densities were as following at 1 day before starting CM preparation for sEV purification; subconfluent for pre-senescent TIG-3 and RPE-1 cells; 8 × 10^2^ cells cm^−2^ for replicative senescent TIG-3 cells; 1 × 10^4^ cells cm^−2^ for Ras-induced senescent TIG-3 cells; 5 × 10^3^ cells cm^−2^ for DXR-induced senescent TIG-3 cells; 1.4 × 10^4^ cells cm^−2^ for DXR-induced senescent RPE-1 cells. The CM to be used for sEV purification was prepared by culturing the cells in DMEM supplemented 5% EV-depleted FBS for 3 days. Replicates are biological replicates (*n*=3). Error bars indicate s.d. *P<0.05 (two-tailed *t*-test).

**Figure 2:**
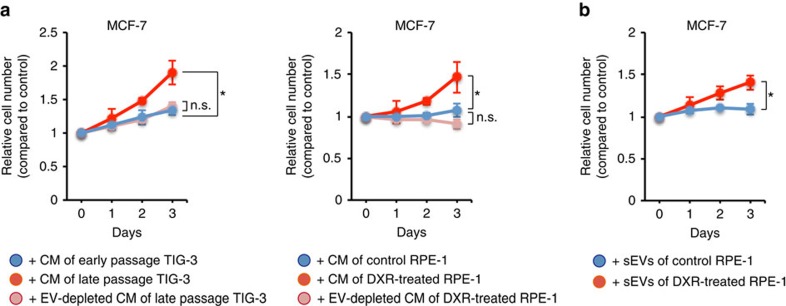
sEVs secreted from senescent cells promote the proliferation of MCF-7 cells. (**a**) Relative numbers of MCF-7 cells grown in the presence of CM compared with the number of cells grown in normal medium (DMEM supplemented with 10% FBS). CM was prepared from control (early passage) and replicative senescent (late passage) TIG-3 cells, or control and DXR-induced senescent RPE-1 cells, and was used directly or after depleting EVs by ultracentrifugation. Cell densities of early and late passage TIG-3 cells were 8 × 10^2^ cells cm^−2^ at 1 day before starting CM preparation. Cell densities of control and DXR-induced senescent RPE-1 cells were 1.4 × 10^4^ cells cm^−2^ at 1 day before starting CM preparation. MCF-7 cells were plated at a density of 4 × 10^2^ cells cm^−2^ 1 day before starting the experiments. The basal proliferation rate, that is, the fold increase in cell number of untreated MCF-7 cells at culture day 3, was 2.03. The CM to be used for cell culture was prepared by culturing the cells in normal medium for 3 days. Statistical analysis was applied only to the data of culture day 3. (**b**) Relative numbers of MCF-7 cells grown in the presence of sEVs prepared from control or DXR-induced senescent RPE-1 cells compared with the number of cells grown in normal medium. Pre-senescent RPE-1 cells were subconfluent and DXR-induced senescent RPE-1 cells were at a density of 1.4 × 10^4^ cells cm^−2^ at 1 day before starting CM preparation. MCF-7 cells were plated at a density of 4 × 10^2^ cells cm^−2^ 1 day before starting the experiments. sEVs were added to the medium at a concentration of 2 × 10^9^ particles per ml. Statistical analysis was applied only to the data of culture day 3. Replicates are biological replicates (*n*=3). Error bars indicate s.d. *P<0.05 (one-way ANOVA with the *post-hoc* Dunnett's two-tailed test for **a** and two-tailed *t*-test for **b**).

Since the SASP is known to be associated with tumour development, depending on the biological contexts^6^,^9^,^13^, we hypothesized that the increased secretion of sEVs from senescent cells may contribute to the pro-tumorigenic effect of the SASP. To explore this possibility, we first examined if the secretion of sEVs is increased by various stresses known to induce cellular senescence. As has been reported for human prostate cancer cells^16^, the secretion of sEVs was significantly increased during cellular senescence in normal human diploid fibroblast (HDF) TIG-3 cells, regardless of how cellular senescence was induced (Fig. 1 and Supplementary Fig. 1a,b). The secretion of sEVs was also increased in human retinal pigment epithelial RPE-1 cells rendered senescent by DNA-damaging agent doxorubicin (DXR; Fig. 1 and Supplementary Fig. 1a,b). Consistent with the pro-tumorigenic effect of the SASP^6^,^9^,^13^, conditioned medium (CM) of replicative senescent TIG-3 cells or DXR-induced senescent RPE-1 cells enhanced the proliferation of human breast cancer MCF-7 cells (Fig. 2a). However, these effects were attenuated when EVs were removed from the CM by ultracentrifugation (Fig. 2a). Knockdown of Rab35, a GTPase that play roles in exosome secretion in RPE-1 cells^28^, also suppressed the pro-proliferative effect of the CM of DXR-induced senescent RPE-1 cells (Supplementary Fig. 1c,d). These results suggest that exosome-like sEVs secreted from senescent cells contribute to the pro-tumorigenic effect of the SASP at least in this experimental setting. To confirm the pro-proliferative effect of sEVs secreted from senescent cells, we next incubated MCF-7 cells with sEVs purified from pre-senescent or senescent cells. Although sEVs purified from DXR-induced senescent RPE-1 cells promoted the proliferation of MCF-7 cells, this was not the case for sEVs purified from pre-senescent RPE-1 cells (Fig. 2b). These results indicate that the qualitative as well as quantitative changes are important for the pro-proliferative effect of sEVs secreted from senescent cells.

### EphA2 is increased in sEVs secreted from senescent cells

**Figure 3:**
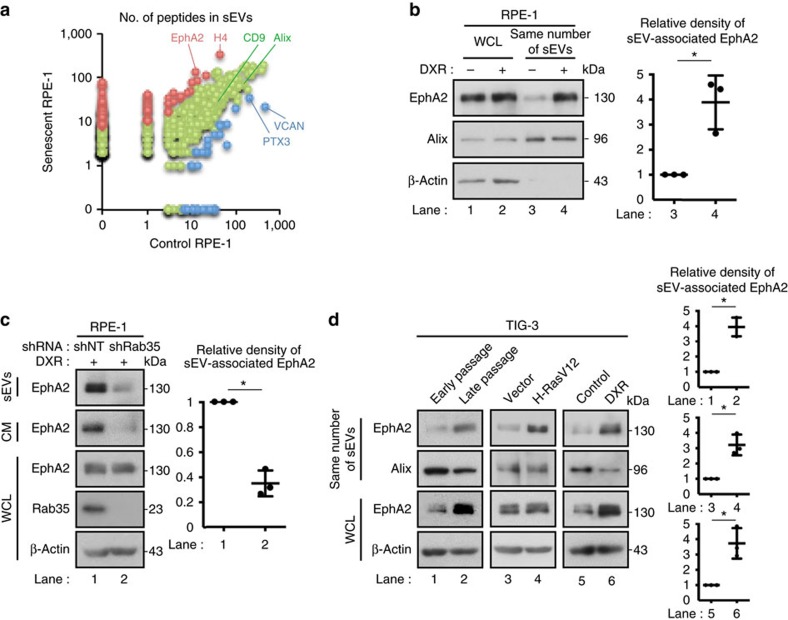
Senescence induces increased secretion of sEV-associated EphA2. (**a**) Comparative proteomic analysis of sEVs secreted from control and DXR-induced senescent RPE-1 cells by using mass spectrometry. The number of peptides detected in sEVs secreted from control and DXR-induced senescent RPE-1 cells are plotted for the identified proteins. Blue and red plots represent the data for the proteins significantly enriched in sEVs secreted from control and DXR-induced senescent RPE-1 cells, respectively. Green plots represent the data for the other proteins. (**b**) Immunoblotting of EphA2, Alix and β-actin in the sEV fraction and WCL of control and DXR-induced senescent RPE-1 cells. The same number of sEVs was loaded in each lane. Dot plot represents the relative density of sEV-associated EphA2 analysed by ImageJ. (**c**) Immunoblotting of indicated proteins in the sEV fraction, CM and WCL of DXR-induced senescent RPE-1 cells expressing non-targeting shRNA (shNT) or Rab35 shRNA (shRab35). Dot plot represents the relative density of sEV-associated EphA2 analysed by ImageJ. (**d**) Immunoblotting of indicated proteins in the sEV fraction and WCL of pre-senescent control and senescent TIG-3 cells. Senescence was induced by serial passage, oncogenic Ras expression or DXR-treatment. The same number of sEVs was loaded in each lane. Dot plots represent the relative density of sEV-associated EphA2 analysed by ImageJ. Replicates are biological replicates (*n*=3). Error bars indicate s.d. *P<0.05 (two-tailed *t*-test).

To identify the underlying mechanisms, we performed a comparative proteomic analysis of sEVs secreted from control and DXR-induced senescent RPE-1 cells. Mass spectrometric analysis identified a total of 678 proteins, including the markers of exosomes/sEVs such as CD9, CD63, CD81, Alix and TSG101 (Fig. 3a and Supplementary Data 1). Organelle markers such as calnexin, calreticulin, Grp78, VDAC and cytochrome *c* were not detected in sEV fractions, suggesting that the contamination of apoptotic bodies was minimal. The most abundant protein enriched in sEVs secreted from DXR-induced senescent RPE-1 cells was histone H4, which might be derived from cytoplasmic chromatin fragments in senescent cells^29^. Interestingly, the second most abundant protein enriched in sEVs secreted from DXR-induced senescent RPE-1 cells was Eph receptor tyrosine kinase EphA2 (Fig. 3a and Supplementary Data 1), which is known to be involved in the regulation of cell proliferation^30^. Immunoblotting confirmed that EphA2 level was higher in sEVs secreted from DXR-induced senescent RPE-1 cells than in the same number of sEVs secreted from pre-senescent RPE-1 cells (Fig. 3b). EphA2 is likely to be associated with exosomes, since Rab35 knockdown drastically decreased EphA2 level in the CM as well as in the sEV fraction prepared from DXR-induced senescent RPE-1 cells (Fig. 3c). Indeed, the amount of EphA2 was much lower in large EVs compared with the sEVs, and the removal of large EVs did not affect the pro-proliferative effect of the CM of DXR-induced senescent RPE-1 cells (Supplementary Fig. 2). sEV-associated EphA2 was also increased in TIG-3 cells rendered senescent by serial passage, oncogenic Ras expression or DXR-treatment (Fig. 3d).

### EphA2 is responsible for pro-proliferative effect of sEVs

**Figure 4:**
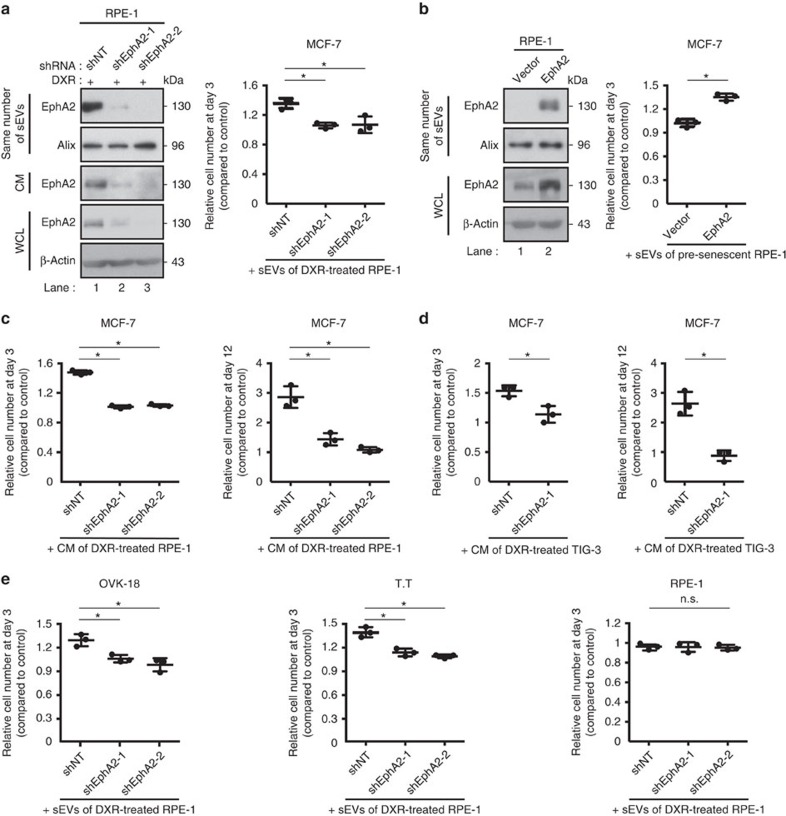
sEV-associated EphA2 promotes cancer cell proliferation. (**a**) Immunoblotting shows successful knockdown of EphA2 in DXR-induced senescent RPE-1 cells. The same number of sEVs was loaded in each lane. Dot plot represents the relative numbers of MCF-7 cells grown for 3 days in the presence of sEVs compared with the number of cells grown for 3 days in normal medium. sEVs were purified from DXR-induced senescent RPE-1 cells expressing non-targeting shRNA (shNT) or EphA2 shRNA (shEphA2-1 or shEphA2-2) and added to the medium at a concentration of 2 × 10^9^ particles per ml. (**b**) Immunoblotting shows successful overexpression of EphA2 in pre-senescent RPE-1 cells. The same number of sEVs was loaded in each lane. Dot plot represents the relative numbers of MCF-7 cells grown for 3 days in the presence of sEVs compared with the number of cells grown for 3 days in normal medium. sEVs were purified from pre-senescent RPE-1 cells expressing empty vector or ectopic EphA2, and added to the medium at a concentration of 2 × 10^9^ particles per ml. (**c**,**d**) Relative numbers of MCF-7 cells grown for 3 and 12 days in the presence of CM prepared from (**c**) DXR-induced senescent RPE-1 cells or (**d**) DXR-induced senescent TIG-3 cells expressing shNT or shEphA2. Normal medium and CM was renewed every 3 days during the long-term (12 days) culture. (**e**) Relative numbers of OVK-18, T.T and pre-senescent RPE-1 cells (plated at a density of 4 × 10^2^ cells cm^−2^ 1 day before starting the experiments) grown for 3 days in the presence of sEVs compared with the number of cells grown for 3 days in normal medium. sEVs were purified from DXR-induced senescent RPE-1 cells expressing shNT or shEphA2 and added to the medium at a concentration of 2 × 10^9^ particles per ml. The basal proliferation rates, that is, the fold increase in cell numbers of untreated OVK-18, T.T and RPE-1 cells at culture day 3, were 2.24, 3.48 and 10.72, respectively. Replicates are biological replicates (*n*=3). Error bars indicate s.d. *P<0.05 (one-way ANOVA with the *post-hoc* Dunnett's two-tailed test for **a**,**c**,**e** and two-tailed *t*-test for **b**,**d**).

To assess the role of EphA2 in the pro-proliferative effect of sEVs secreted from senescent cells, we incubated MCF-7 cells with sEVs purified from DXR-induced senescent RPE-1 cells expressing control or EphA2 shRNA. Notably, EphA2 knockdown abolished the pro-proliferative effect of sEVs secreted from DXR-induced senescent RPE-1 cells (Fig. 4a), and reintroduction of shRNA-resistant EphA2 cDNA restored their pro-proliferative effect (Supplementary Fig. 3a). Conversely, EphA2 overexpression was sufficient to render sEVs secreted from pre-senescent RPE-1 cells pro-proliferative (Fig. 4b). Furthermore, EphA2 knockdown abolished the pro-proliferative effect of the CM of DXR-induced senescent cells in both RPE-1 and TIG-3 cells (Fig. 4c,d). These results, together with the observation that EphA2 depletion had no effect on the secretion of sEVs (Supplementary Fig. 3b,c), indicate that EphA2 plays a key role in the pro-proliferative effect of sEVs secreted from senescent cells, at least in this experimental setting. Interestingly, recombinant EphA2 did not promote MCF-7 cell proliferation (Supplementary Fig. 3d), suggesting that soluble and sEV-associated EphA2 have different functions, as has been reported for TGF-β^31^.

### sEV-associated EphA2 induces EphA2/ephrin-A1 reverse signal

**Figure 5:**
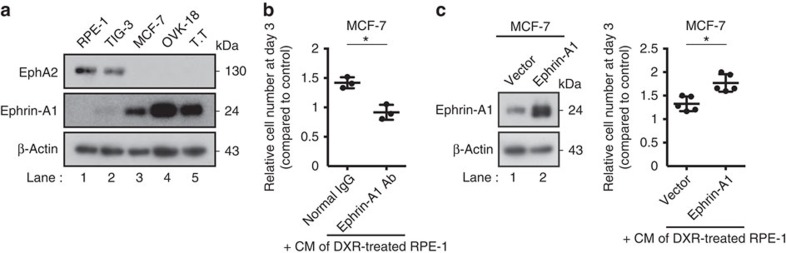
Ephrin-A1 mediates the growth-promoting effect of sEV-associated EphA2. (**a**) Immunoblotting of EphA2, ephrin-A1 and β-actin in the WCL of indicated cell lines. (**b**) Relative numbers of MCF-7 cells grown for 3 days in the presence of CM compared with the number of cells grown for 3 days in normal medium. Normal rabbit IgG or rabbit anti-ephrin-A1 IgG (AER-031; Alomone) was added to the medium at a concentration of 5 μg ml^−1^. CM was prepared from DXR-induced senescent RPE-1 cells. (**c**) Immunoblotting shows successful overexpression of ephrin-A1 in MCF-7 cells. Dot plot represents the relative numbers of MCF-7 cells grown for 3 days in the presence of CM compared with the number of cells grown for 3 days in normal medium. Empty vector or ectopic ephrin-A1 was expressed in MCF-7 cells. CM was prepared from DXR-induced senescent RPE-1 cells. Replicates are biological replicates (*n*=3 for **b** and *n*=5 for **c**). Error bars indicate s.d. *P<0.05 (two-tailed *t*-test).

sEVs secreted from DXR-induced senescent RPE-1 cells also promoted the growth of OVK-18 cells (human ovarian cancer cell line) and T.T cells (human oesophageal cancer cell line) in an EphA2-dependent manner (Fig. 4e). However, the growth of pre-senescent RPE-1 cells was not affected by sEVs secreted from DXR-induced senescent RPE-1 cells (Fig. 4e). These results are consistent with previous reports showing that the SASP does not promote the proliferation of non-cancerous cells^10^,^32^. Since ephrin-A1, a major ligand of EphA2, is known to be upregulated in many types of cancer cells^30^, we hypothesized that ephrin-A1 mediates the pro-proliferative effect of sEV-associated EphA2 in cancer cells used in this study. Indeed, ephrin-A1 levels were higher in cancerous MCF-7, OVK-18 and T.T cells than in non-cancerous RPE-1 and TIG-3 cells (Fig. 5a). Ephrin-A1 antibody, whose epitope overlaps with the ephrin receptor-binding domain of ephrin-A1 and can interfere with the binding of EphA2 to ephrin-A1 (Supplementary Fig. 4a), suppressed the effect of senescent cell CM on MCF-7 cell proliferation (Fig. 5b and Supplementary Fig. 4b–d). It is possible, however, that the suppressive effect of ephrin-A1 antibody is due to inhibition of ephrin-A1 binding to other proteins such as co-receptors that are required to produce reverse signal. In addition to this result, the pro-proliferative effect of the CM of DXR-induced senescent RPE-1 cells was enhanced when ephrin-A1 was overexpressed in MCF-7 cells (Fig. 5c). These results indicate that sEVs secreted from the senescent cells promote cell proliferation at least partially through EphA2/ephrin-A1 signalling in MCF-7 cells.

**Figure 6:**
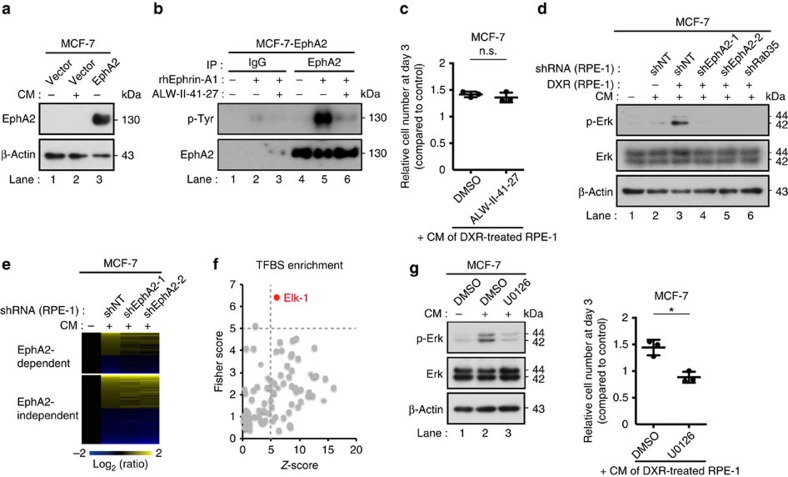
sEV-associated EphA2 promotes cancer cell proliferation through EphA2/ephrin-A1 reverse signalling. (**a**) Immunoblotting of EphA2 and β-actin in the WCL of MCF-7 cells expressing empty vector or ectopic EphA2. Cells were grown for 3 days with or without CM prepared from DXR-induced senescent RPE-1 cells. (**b**) EphA2 immunoprecipitates prepared from MCF-7 cells expressing ectopic EphA2 were immunoblotted with anti-phosphotyrosine and anti-EphA2 antibody. The indicated samples were treated with recombinant human ephrin-A1 (500 ng ml^−1^) for 20 min. Eight-hour pre-treatment with the EphA2 inhibitor ALW-II-41-27 (100 nM) suppressed ephrin-A1-induced EphA2 phosphorylation. (**c**) Relative numbers of MCF-7 cells grown for 3 days in the presence of CM compared with the number of cells grown for 3 days in normal medium. DMSO or ALW-II-41-27 (100 nM) was added to the medium. (**d**) Immunoblotting of phospho-Erk, total-Erk and β-actin in the WCL of MCF-7 cells grown for 3 days with or without CM prepared from control or DXR-induced senescent RPE-1 cells expressing non-targeting shRNA (shNT), shEphA2 or shRab35. (**e**) The heatmap shows the relative gene expression levels in MCF-7 cells grown for 3 days with or without CM prepared from DXR-induced senescent RPE-1 cells expressing shNT or shEphA2. Genes whose expression levels were changed more than 1.2-fold by the CM of shNT-expressing cells are shown. (**f**) EphA2-dependent upregulated genes were analysed with oPOSSUM-3. Each circle in the plot shows an enrichment of the targets of a different transcription factor. (**g**) Immunoblotting of phospho-Erk, total-Erk and β-actin in the WCL of MCF-7 cells grown for 3 days with or without CM. Dot plot represents the number of MCF-7 cells grown for 3 days in the presence of CM compared with the number of cells grown for 3 days in normal medium. DMSO or U0126 (500 nM) was added to the medium. CM was prepared from DXR-induced senescent RPE-1 cells. Replicates are biological replicates (*n*=3). Error bars indicate s.d. *P<0.05 (two-tailed *t*-test).

Eph receptor–ephrin complexes are known to emanate bidirectional signals: forward signals that propagate in Eph-expressing cells through Eph kinase activity, and reverse signal that propagate in ephrin-expressing cells^30^. Eph receptor on sEVs may activate forward signals after incorporated into recipient cells, or may otherwise directly activate reverse signal in recipient cells as has been reported for EphB2 (ref. 33). Incubating MCF-7 cells in the CM of DXR-induced senescent RPE-1 cells did not cause a noticeable increase in intracellular EphA2 levels, suggesting that the incorporation of sEV-associated EphA2 was minimal (Fig. 6a). Moreover, the EphA2 tyrosine kinase inhibitor ALW-II-41-27 (Fig. 6b) did not substantially suppress the pro-proliferative effect of the CM of DXR-induced senescent RPE-1 cells (Fig. 6c). These data strongly suggest that EphA2/ephrin-A1 reverse signalling is involved in the pro-proliferative effect of sEVs secreted from senescent cells. Consistent with the previous findings of Erk activation downstream of Eph receptor–ephrin reverse signalling^30^, Erk phosphorylation was found to be enhanced when MCF-7 cells were incubated in the CM of DXR-induced senescent RPE-1 cells (Fig. 6d). This was not the case when EphA2 or Rab35 was knocked down in DXR-induced senescent RPE-1 cells (Fig. 6d). To further explore the downstream effects of sEV-associated EphA2, we performed microarray analysis and compared the gene expression profiles of MCF-7 cells incubated in normal medium or in the CM of DXR-induced senescent RPE-1 cells expressing control or EphA2 shRNA. Gene expression changes induced in MCF-7 cells by CM treatment were partially suppressed by knocking down EphA2 in DXR-induced senescent RPE-1 cells (Fig. 6e). Remarkably, two different bioinformatic programs, oPOSSUM-3 (ref. 34) and g:Profiler^35^, both showed that Elk-1, a key downstream regulator of the Erk pathway, was the only transcription factor whose targets were enriched in the genes upregulated by CM in an EphA2-dependent manner (Fig. 6f). These results, in conjunction with the observation that Erk inhibition by the MEK inhibitor U0126 abolished the pro-proliferative effect of the CM (Fig. 6g), lead us to propose that sEV-associated EphA2 promotes the proliferation of ephrin-A1-expressing cancer cells by activating the Erk pathway through EphA2/ephrin-A1 reverse signalling.

### sEV sorting of EphA2 is facilitated by its phosphorylation

**Figure 7:**
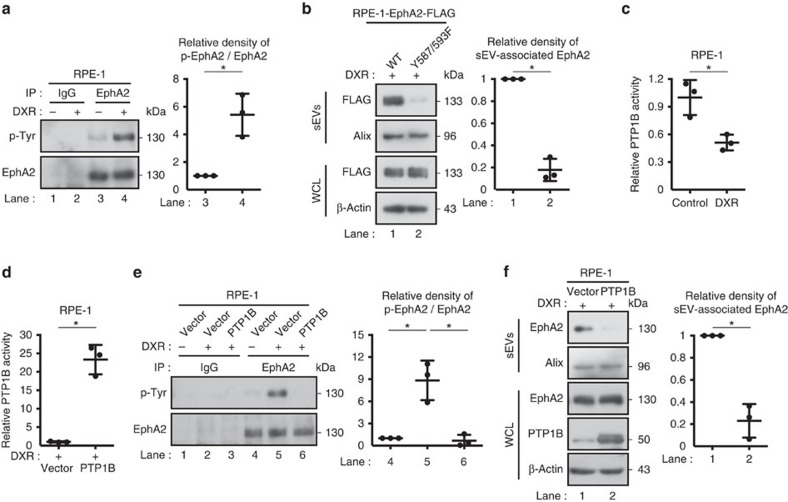
EphA2 phosphorylation facilitates its sorting into sEVs in senescent cells. (**a**) EphA2 immunoprecipitates prepared from control and DXR-induced senescent RPE-1 cells were immunoblotted with anti-phosphotyrosine and anti-EphA2 antibody. Dot plot represents the relative density of phospho-EphA2/EphA2 analysed by ImageJ. (**b**) Immunoblotting of indicated proteins in the sEV fraction and WCL of DXR-induced senescent RPE-1 cells expressing wild-type or phospho-resistant EphA2 tagged with FLAG. Dot plot represents the relative density of sEV-associated EphA2 analysed by ImageJ. (**c**) Relative PTP1B activity in control and DXR-induced senescent RPE-1 cells. (**d**) Relative PTP1B activity in DXR-induced senescent RPE-1 cells expressing empty vector or ectopic PTP1B. (**e**) Immunoprecipitates prepared from control and DXR-induced senescent RPE-1 cells expressing empty vector or ectopic PTP1B were immunoblotted with anti-phosphotyrosine and anti-EphA2 antibody. Dot plot represents the relative density of phospho-EphA2/EphA2 analysed by ImageJ. (**f**) Immunoblotting of indicated proteins in the sEV fraction and WCL of DXR-induced senescent RPE-1 cells expressing empty vector or ectopic PTP1B. Dot plot represents the relative density of sEV-associated EphA2 analysed by ImageJ. Replicates are biological replicates (*n*=3). Error bars indicate s.d. *P<0.05 (two-tailed *t*-test for **a**–**d**,**f** and one-way ANOVA with the *post-hoc* Dunnett's two-tailed test for **e**).

We next investigated the mechanisms for the enrichment of EphA2 in sEVs secreted from senescent cells. Because intracellular levels of EphA2 were unchanged during DXR-induced senescence in RPE-1 cells (Fig. 3b), we considered that some types of post-translational modifications of EphA2 may be involved in the enrichment of EphA2 in sEVs secreted from senescent cells. As with many other receptor tyrosine kinases, auto-phosphorylation of EphA2 promotes its endocytic internalization^36^, which is the first step of its exosomal sorting. Therefore, we examined tyrosine phosphorylation of EphA2 in senescent cells. Notably, tyrosine phosphorylation of EphA2 was increased not only in DXR-induced senescent RPE-1 cells but also in senescent TIG-3 cells, regardless of how cellular senescence was induced (Fig. 7a and Supplementary Fig. 5a). Although ectopically expressed FLAG-tagged wild-type EphA2 was incorporated into the sEVs of DXR-induced senescent RPE-1 cells, this was not the case if two tyrosine residues (Tyr 587 and Tyr 593) of EphA2, whose phosphorylation is critical for EphA2 tyrosine kinase activity^37^, were mutated to phenylalanine (Fig. 7b). These results suggest that enhanced tyrosine phosphorylation of EphA2 is involved in its sEV sorting in senescent cells.

### ROS induce EphA2 phosphorylation via PTP1B inactivation

**Figure 8:**
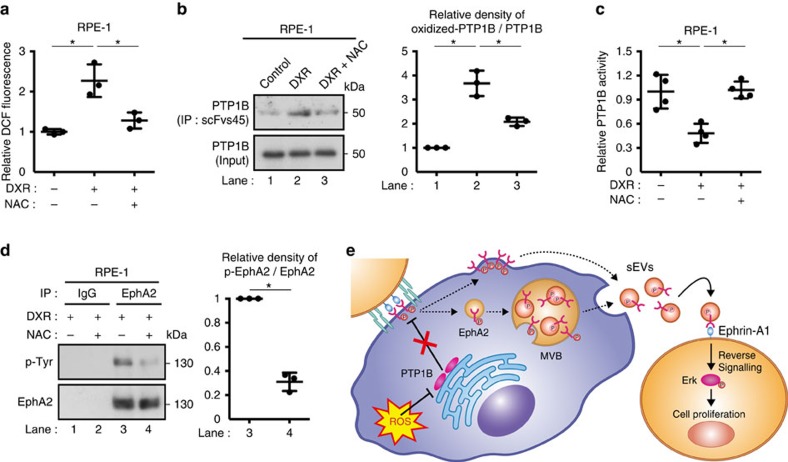
Increased ROS induce EphA2 phosphorylation via PTP1B inactivation. (**a**) Intracellular ROS levels were measured by DCF fluorescence in RPE-1 cells treated with or without DXR and NAC. NAC was added to the medium 6 h before analysis at a concentration of 2 mM. (**b**) Oxidized-PTP1B was immunoprecipitated by scFvs45 antibody from RPE-1 cells treated with or without DXR and NAC. Inputs and immunoprecipitates were immunoblotted with anti-PTP1B antibody. Dot plot represents the relative density of oxidized-PTP1B/PTP1B analysed by ImageJ. (**c**) Relative PTP1B activity in RPE-1 cells treated with or without DXR and NAC. (**d**) EphA2 immunoprecipitates prepared from RPE-1 cells treated with or without DXR and NAC were immunoblotted with anti-phosphotyrosine and anti-EphA2 antibody. Dot plot represents the relative density of phospho-EphA2/EphA2 analysed by ImageJ. (**e**) Schematic model of the regulation and function of sEV-associated EphA2 in senescent cells. Replicates are biological replicates (*n*=3 for **a**,**b**,**d** and *n*=4 for **c**). Error bars indicate s.d. *P<0.05 (one-way ANOVA with the *post-hoc* Dunnett's two-tailed test for **a**–**c**) and two-tailed *t*-test for **d**).

The above findings led to questions regarding the mechanisms underlying the enhanced tyrosine phosphorylation of EphA2 in senescent cells. In TIG-3 cells rendered senescent by serial passage or DXR-treatment, increased expression of EphA2 may be one of the causes of EphA2 phosphorylation, since overexpression of receptor tyrosine kinase can increase the chance of ligand-independent dimerization^38^. Indeed, overexpression of EphA2 was enough to induce its tyrosine phosphorylation in pre-senescent RPE-1 cells (Supplementary Fig. 5b). However, intracellular levels of EphA2 were not increased in DXR-induced senescent RPE-1 cells and Ras-induced senescent TIG-3 cells (Fig. 3b,d), suggesting that there should be another mechanism of EphA2 phosphorylation in senescent cells. Considering that at least ephrin-A1 levels remained very low in DXR-induced senescent RPE-1 cells (Supplementary Fig. 5c), we speculated that the enhanced tyrosine phosphorylation of EphA2 might be due to decreased phosphatase activity in DXR-induced senescent RPE-1 cells. As expected, the activity of PTP1B, a protein tyrosine phosphatase that dephosphorylates EphA2 (ref. 39), was substantially decreased in DXR-induced senescent RPE-1 cells (Fig. 7c). This is interesting because recent study has shown that PTP1B is critical for suppressing ligand-independent auto-phosphorylation of EphA2 (ref. 40). Ectopic PTP1B expression suppressed tyrosine phosphorylation of EphA2 and inhibited its sEV sorting in DXR-induced senescent RPE-1 cells (Fig. 7d-f). Conversely, the PTP1B inhibitor (CAS:765317-72-4) enhanced tyrosine phosphorylation of EphA2 and promoted its sEV sorting in pre-senescent RPE-1 cells (Supplementary Fig. 5d,e). These results indicate that the reduced PTP1B activity contributes to the increased sEV sorting of EphA2 in DXR-induced senescent RPE-1 cells. It is known that the activity of PTP1B is attenuated when its catalytic cysteine residue is oxidized by reactive oxygen species (ROS), which are commonly increased in senescent cells^5^,^41^,^42^. Therefore, we tested whether increased ROS levels are involved in EphA2 tyrosine phosphorylation in senescent cells. In DXR-induced senescent RPE-1 cells, ROS levels and PTP1B oxidation were both increased (Fig. 8a,b). Reduction of ROS levels by antioxidant N-acetyl-cysteine (NAC)-suppressed PTP1B oxidation and restored its activity in DXR-induced senescent RPE-1 cells (Fig. 8a–c). Moreover, these effects of NAC were accompanied by suppression of EphA2 tyrosine phosphorylation (Fig. 8d).

## Discussion

Collectively, our data support a model in which increased ROS levels enhance EphA2 phosphorylation by inactivating PTP1B and thereby promote the sorting of EphA2 into sEVs in DXR-induced senescent cells. Increased expression of EphA2 might be also important for the phosphorylation and sEV sorting of EphA2 in some types of senescent cells. Although our data suggest that the majority of EphA2-containing sEVs are exosomes, we could not exclude the possibility that EphA2 is also secreted via other types of sEVs. Once released, sEV-associated EphA2 promotes cancer cell proliferation by activating the Erk pathway through EphA2/ephrin-A1 reverse signalling (Fig. 8e). However, it should be noted that our data do not exclude the possibility that other types of ephrin-A ligands can also mediate the pro-proliferative effect of sEV-associated EphA2 secreted from senescent cells.

**Figure 9:**
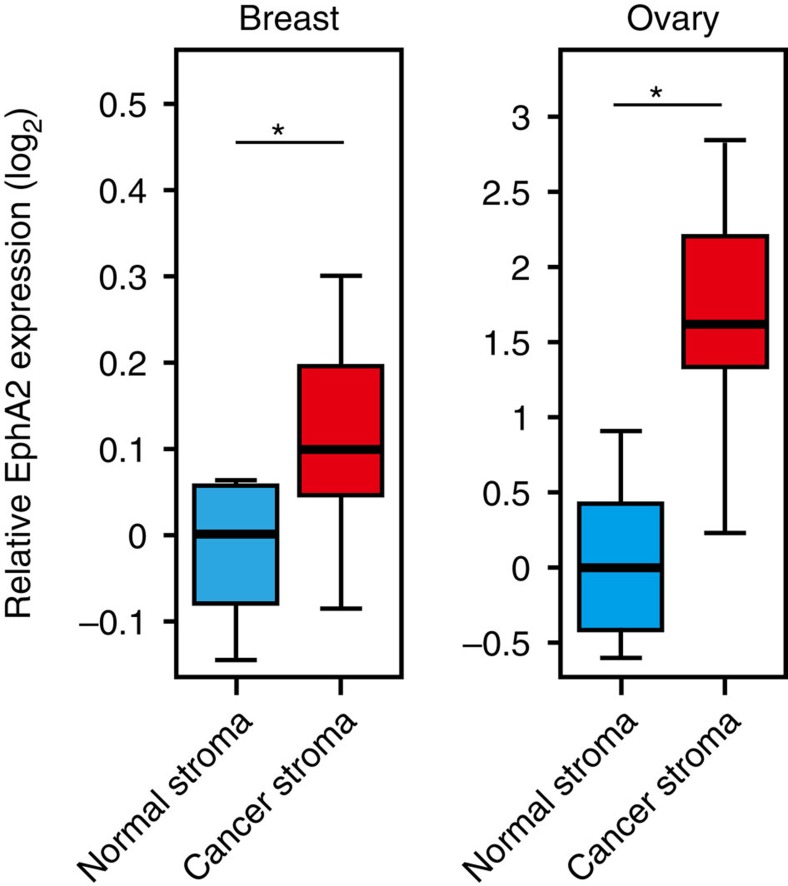
EphA2 expression is increased in the stroma of breast and ovarian cancers. The left boxplot shows EphA2 mRNA expression levels in normal breast stroma (*n*=6) and breast cancer stroma (*n*=26). The right boxplot shows EphA2 mRNA expression levels in normal ovarian stroma (*n*=8) and ovarian cancer stroma (*n*=31). *P<0.05 (Wilcoxon test).

Although other components of sEVs secreted from senescent cells may have important roles in other biological functions, our data show that EphA2 is at least in part responsible for the pro-proliferative effect of sEVs secreted from senescent cells. Interestingly, we found that expression levels of EphA2 is increased in the stroma of human breast and ovarian cancer as compared to normal stromal tissues (Fig. 9), by using published microarray data^43^,^44^. Thus, it is tempting to speculate that senescence-associated secretion of sEV-associated EphA2 may contribute to the age-related increase in cancer incidence. Our findings on the mechanism of cell context-dependent cargo sorting into sEVs may open up new possibilities to control harmful side effects of senescent cell secretome.

## Methods

### Chemicals

DXR was from Wako. The recombinant human EphA2 was from R&D Systems (3035-A2-100). The recombinant human ephrin-A1 was from ThermoFisher Scientific (10882-H08H). ALW-II-41-27 was from MedChem Express. U0126 and NAC were from Sigma, and the PTP1B inhibitor (CAS:765317-72-4) was from Millipore.

### Cell culture

RPE-1, TIG-3, MCF-7, T.T and OVK-18 cells were cultured in DMEM supplemented with 10% fetal bovine serum (FBS). TIG-3 and OVK-18 cells were from Japanese Cancer Research Resources Bank (JCRB). RPE-1, MCF-7 and T.T cells were from Clontech, ATCC and RIKEN BioResource Center, respectively. The passage numbers of TIG-3 cells used in this study was <52, excluding replicative senescent cells. All cell lines were routinely tested for mycoplasma infection and were found to be negative.

### Senescence induction

Replicative senescence was induced in TIG-3 cells by serial passage. The cell density of replicative senescent TIG-3 cells was 8 × 10^2^ cells cm^−2^ when preparing CM. To induce senescence by oncogenic Ras expression, TIG-3 cells were infected with a retrovirus encoding puromycin resistance and H-Ras^G12V^, and were cultured with 1 μg ml^−1^ puromycin for 10 days. The cell density of Ras-induced senescent TIG-3 cells was 1 × 10^4^ cells cm^−2^ when preparing CM. To induce senescence by DXR, TIG-3 and RPE-1 cells were cultured in medium containing DXR at concentrations of 250 and 150 ng ml^−1^ for 10 and 4 days, respectively. One day before DXR-treatment, TIG-3 and RPE-1 cells were plated at a density of 5 × 10^3^ and 1.4 × 10^4^ cells cm^−2^, respectively, and were not passaged after the DXR-treatment.

### SA-β-gal assay

SA-β-gal assay was performed as described previously^45^. Cells were fixed in fixation buffer (2% PFA and 0.2% glutaraldehyde in PBS) and stained with staining solution (5 mM potassium ferricyanide, 5 mM potassium ferrocyanide, 2 mM MgCl_2_, 150 mM NaCl, 1 mg ml^−1^ X-Gal) in citrate/sodium phosphate buffer (pH 6) overnight at 37 °C. Cells were then washed twice with PBS, and the percentage of stained cells was determined.

### CM preparation

For sEV purification, CM was prepared by incubating cells for 3 days in DMEM supplemented with 5% FBS depleted of sEVs by ultracentrifugation at 100,000*g* for 16 h. Pre-senescent control cells were plated at subconfluent density 1 day before starting CM preparation for sEV purification. The CM to be used for cell culture was prepared by incubating cells for 3 days in DMEM supplemented with 10% FBS. Pre-senescent control cells were plated at the same density as their senescent counterparts 1 day before starting CM preparation for use in cell culture. The CM was filtered through a 0.22-μm filter, diluted 1:1 with fresh medium and then used for cell culture. This filtration process was skipped in the experiments presented in Supplementary Fig. 2b. In the experiment in which CM was depleted of sEVs, the CM was centrifuged at 100,000*g* for 16 h before diluting with fresh medium. The cells to be analysed at days 3 and 12 after starting treatment with CM or sEVs were plated at densities of 400 and 100–200 cells cm^−2^, respectively, and were cultured in medium without phenol red throughout the treatment.

### sEV isolation

sEVs were purified by differential centrifugation as previously described^46^, with some modifications. The CM was first centrifuged at 10,000*g* for 30 min. The supernatant was filtered through a 0.22-μm filter, layered on a 30% sucrose/D_2_O cushion and centrifuged at 100,000*g* for 70 min. The sucrose cushion was collected and washed twice with PBS by centrifugation at 100,000*g* for 3 h. The pelleted sEVs were then re-suspended in PBS. All centrifugations were carried out at 4 °C. The size and number of sEVs were determined using the LM10 nanoparticle characterization system (NanoSight).

### Plasmids

All shRNA constructs were cloned into the pRetro-Super retroviral vector^47^. The sequence of each shRNA was as follows: shNT (non-targeting control shRNA), 5′-CATTGCTATAGAGGCAGAT-3′; shEphA2-1, 5′-CTATTCTGTCAGTGTTAAA-3′; shEphA2-2, 5′-GGATAAGTTTCTATTCTGT-3′; and shRab35, 5′-GCGGTGGCTTCACGAAATCAACC-3′. Constructs expressing EphA2, ephrin-A1 or PTP1B were prepared by cloning the corresponding cDNA into the MaRX retroviral vector^48^. Rescue of EphA2 expression in cells expressing EphA2 3′UTR shRNA (shEphA2-1) was performed using an EphA2 cDNA-expressing construct. To prepare shRNA-resistant Rab35, 4 point mutations, which do not change amino acids, were introduced in the shRab35 target sequence by using the following primers: 5′-ACGAGATAAACCAGAACTGTGATGATGTGTGCC-3′ and 5′-GTAGCCAGCGCTTGACGTTGACAAAGGACTCGGC-3′. Wild-type and phospho-resistant EphA2 (Y587F/Y593F) tagged with C-terminal 3xFLAG were also expressed in the MaRX retroviral vector. The primers used for preparing phospho-resistant EphA2 were as follows: 5′-CCCACACATTTGAGGACCCCAACCAGGCTG-3′ and 5′-GGTCCACGAATGTCTTCAGGGGCTTCAGTTG-3′. For H-Ras^G12V^ expression, the pBabe retroviral vector was used. Retroviral supernatants were prepared by transfecting the packaging cell line LinXE or LinXA with the retroviral vectors. After retroviral infection, RPE-1, TIG-3 and MCF-7 cells were selected using 2, 1 and 0.5 μg ml^−1^ puromycin, respectively.

### Immunoblotting

Cells and sEVs were lysed in RIPA buffer (50 mM Tris pH 8, 150 mM NaCl, 1% NP-40, 0.5% sodium deoxycholate, 0.1% SDS, 1 mM NaF, 0.1 mM sodium orthovanadate) supplemented with protease inhibitor cocktail (cat. no., 25955; Nacalai Tesque). Lysates and immunoprecipitates were denatured in Laemmli buffer containing DTT and 2-mercaptoethanol, respectively. The denaturation was performed for 2 h at 37 °C for the lysates of EVs and for 5 min at 100 °C for the other samples. After cooling, the proteins were separated by SDS-PAGE, transferred onto PVDF membranes, blocked with 5% milk or 2% BSA for 1 h and probed with following primary antibodies: anti-Alix (12422-1-AP; Proteintech), anti-β-actin (clone AC-74; SIGMA), anti-CD9 (ab92726; Abcam), anti-ephrin-A1 (AER-031; Alomone Labs), anti-EphA2 (C-20; Santa Cruz), anti-Erk (#9102; CST), anti-FLAG (PM020; MBL), anti-p21 (C-19; Santa Cruz), anti-phospho-Erk (E-4; Santa Cruz), anti-phospho-p53 (Ser15) (#9284; CST), anti-phosphotyrosine (clone 4G10; Millipore), anti-PTP1B (clone FG6-1G; Millipore) and anti-Rab35 (11329-2-AP; Proteintech). All primary antibodies were diluted to 1 μg ml^−1^. After washing, membranes were incubated with secondary antibodies (GE Healthcare) (1:1,000) for 1 h at room temperature (RT) and visualized with enhanced chemiluminescence reagents. Uncropped gel images are provided in Supplementary Figs 6–12.

### Mass spectrometric analysis

sEVs were lysed in RIPA buffer supplemented with protease inhibitor cocktail. Lysates were denatured for 2 h at 37 °C in Laemmli buffer containing DTT. After cooling, the proteins were resolved by SDS-PAGE, following which the gel was stained with SYPRO Ruby (BioRad). After visualization, each lane of the gel was cut into pieces and subjected to trypsin digestion and liquid chromatography tandem-mass spectrometry (LC–MS/MS) analysis (LTQ Orbitrap Velos, ThermoScientific). The mass spectral data were analysed using the Scaffold software (version 3.4.5, Proteome Software). A protein threshold of 99%, minimum of two peptides, and a peptide threshold of 95% were used as the cut-off values. Proteins identified with >5-fold difference in peptide number between sEVs secreted from control and DXR-induced senescent RPE-1 cells, with a *P* value of <0.01 (Fisher's exact test), were considered as significantly enriched proteins.

### ELISA binding assay of rmEphrin-A1 to immobilized rmEphA2

ELISA assays were performed as previously described^49^, with some modifications. Briefly, 96-well ELISA high binding plates (cat. no., 2592; Costar) were incubated overnight at 4 °C with 100 μl per well of 1 μg ml^−1^ recombinant mouse EphA2-Fc (639-A2; R&D Systems) diluted in PBS. The day after, wells were washed with washing buffer (PBS+0.05% Tween-20) and blocked with blocking solution (PBS+0.5% BSA) for 2 h at 37 °C. Biotinylated recombinant mouse ephrin-A1-Fc (BT602; R&D Systems) (5 ng ml^−1^) was pre-incubated with normal rabbit IgG or rabbit anti-ephrin-A1 IgG (100 μg ml^−1^) for 1 h at 37 °C, and then added to the wells (100 μl per well) and incubated for 1 h at 37 °C. The wells were washed and incubated with 100 μl per well Streptavidin-HRP (S5512; Sigma) in blocking solution (0.05 μg ml^−1^) for 20 min at RT, and then washed again and incubated at RT with 0.1 mg ml^−1^ tetra-methylbenzidine (T2885; Sigma) reconstituted in peroxide buffer (50 mM citrate-phosphate buffer, pH 5+0.02% H_2_O_2_). The reaction was stopped with 3 N HCl 100 μl per well, and the absorbance at 450 nm was measured.

### Immunoprecipitation

For EphA2 immunoprecipitation, the cells were lysed in Triton buffer (50 mM Tris pH 7.5, 150 mM NaCl, 1 mM EDTA, 0.5% Triton, 1 mM NaF, 0.1 mM sodium orthovanadate) supplemented with protease inhibitor cocktail. EphA2 was immunoprecipitated from 150 μg of lysates with 1 μg of anti-EphA2 antibody (C-20; Santa Cruz) by using Dynabeads Protein G magnetic beads (Dynal) according to the manufacturer's instructions. Immunoprecipitates were eluted with Laemmli buffer for 10 min at 70 °C. For the immunoprecipitation of oxidized-PTP1B, cells were lysed in degassed IP buffer (50 mM HEPES pH 7.5, 150 mM NaCl, 5 mM EDTA, 10% glycerol, 1% Triton) supplemented with protease inhibitor cocktail under anaerobic conditions. Five micrograms of anti-oxidized-PTP1B antibody (scFv45; Millipore), 10 mM imidazole and 0.05% BSA were added to lysates containing 500 μg protein, and the mixture was incubated overnight at 4 °C. Subsequently, Ni-NTA agarose (ThermoFisher Scientific) was added to the mixture, which was then incubated for 1 h at 4 °C. After washing with the washing buffer (20 mM HEPES pH 7.5, 300 mM NaCl, 20 mM imidazole, 0.05% Tween-20, 0.05% BSA), precipitates were eluted with the elution buffer (20 mM HEPES pH 7.5, 300 mM NaCl, 500 mM Imidazole, 0.05% Tween-20) for 15 min at 4 °C.

### Microarray analysis

Microarray experiments were conducted using SurePrint G3 Human GE microarray 8 × 60K ver 2.0 (Agilent). cDNA labelling, hybridizations and scanning were performed by DNA ChiP Research (Japan), an Agilent-certified service provider. The expression values for each array were multiplied to obtain 75th percentiles equal to 250. Expression values lower than 50 were then set to 50. Among the genes whose expression levels were changed more than 1.2-fold by CM treatment, those whose differential expression was reduced by both shEphA2-1 and shEphA2-2 shRNA were classified as EphA2-dependent genes. Transcription factor binding site enrichment analysis were performed using oPOSSUM-3 (ref. 34) and g:Profiler^35^. The parameters used in the oPOSSUM-3 were as follows: conservation cut-off=0.4, matrix score threshold=80%, analysed sequences=5 kb upstream of transcription start sites. All expressed genes (genes whose expression values were >50 in at least one of the samples) were used as the background gene set in both programs. In the case of clinical samples^43^,^44^, normalized microarray data were retrieved from GEO (accession numbers: GSE482335 and GSE4059536).

### PTP1B activity assay

Cells were lysed in degassed IP buffer (50 mM Tris pH 7.4, 5 mM EDTA, 150 mM NaCl, 1% Triton) supplemented with protease inhibitor cocktail under anaerobic conditions. PTP1B was immunoprecipitated from 200 μg of lysates with 1 μg of anti-PTP1B antibody (AE-2 J; Millipore), using Protein G magnetic beads (Dynal) according to the manufacturer's instructions. The beads were then washed three times in the IP buffer and once in assay buffer (50 mM HEPES pH 7, 100 mM NaCl, 0.1% BSA) and incubated with 20 mM DiFMUP (ThermoFisher Scientific) in the assay buffer for 20 min at RT. After incubation, DiFMU fluorescence, which reflects PTP1B activity, was measured with excitation at 358 nm and emission at 455 nm.

### Intracellular ROS quantification

Intracellular ROS levels were quantified using the fluorescent probe H_2_DCFDA. Cells were incubated with 5 mM H_2_DCFDA (ThermoFisher Scientific) for 20 min at 37 °C. After a wash, DCF fluorescence was measured with excitation at 485 nm and emission at 530 nm. The DCF fluorescence levels, reflecting the relative ROS levels, were normalized with respect to the total amount of protein.

### Statistical analysis

All quantitative data are presented as mean±s.d. values, except for those represented in boxplots. Two-tailed Student's *t*-test and one-way ANOVA with the *post-hoc* Dunnett's two-tailed test were used to assess statistical significance, unless otherwise indicated.

### Data availability

Microarray data have been deposited in the Gene Expression Omnibus (GEO) database (http://www.ncbi.nlm.nih.gov/geo/) under the accession number GSE81373.

## Additional information

**How to cite this article:** Takasugi, M. *et al*. Small extracellular vesicles secreted from senescent cells promote cancer cell proliferation through EphA2. *Nat. Commun.* **8,** 15728 doi: 10.1038/ncomms15728 (2017).

**Publisher's note:** Springer Nature remains neutral with regard to jurisdictional claims in published maps and institutional affiliations.

## Supplementary Material

Supplementary Information Supplementary Figures

Supplementary Data 1 Lists of proteins identified in control and senescent cell sEVs
