# Supplementary material for: Small extracellular vesicles secreted from senescent cells promote cancer cell proliferation through EphA2
Source: Nat Commun. 2017 Jun 6;8:15729. doi: 10.1038/ncomms15728 (PMC5467215; doi:10.1038/ncomms15728)
Supplement: Supplementary Information — Supplementary Figures [file ncomms15728-s1.pdf]

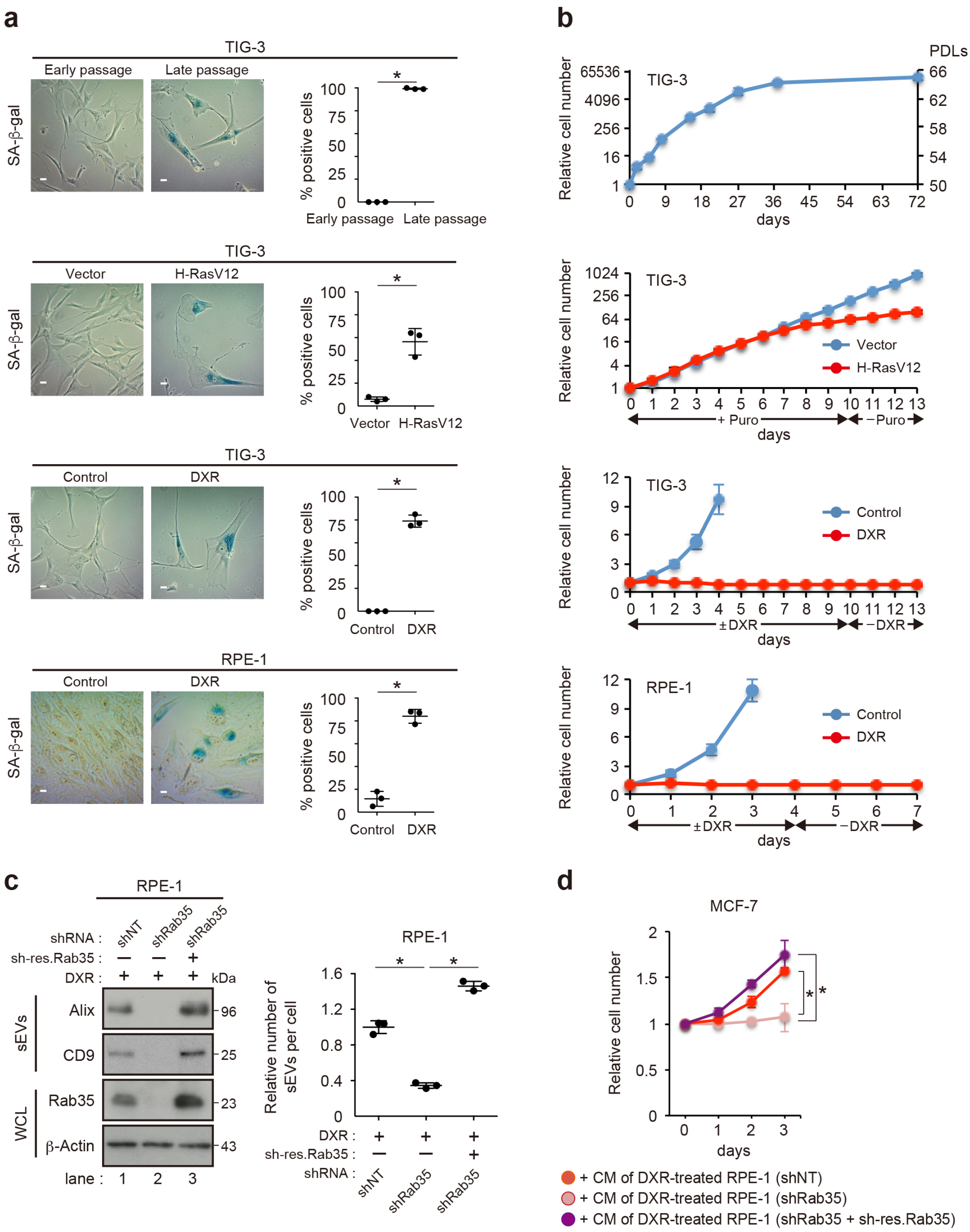

Supplementary Figure 1 | Rab35 knockdown abolishes the pro-proliferative effect of CM of DXR-induced senescent RPE-1 cells. (**a**) SA-β-gal staining of pre-senescent control and senescent cells. Senescence was induced by serial passage, oncogenic Ras expression, or DXR treatment in TIG-3 or RPE-1 cells. Representative images are shown. Dot plots show the percentages of the SA-β-gal positive cells. One hundred cells were counted in each group. Scale bars, 10 µm. (**b**) Growth curves of control and senescent cells. (**c**) Immunoblotting of Alix and CD9 in the sEV fraction and of Rab35 and β-actin in the WCL of DXR-induced senescent RPE-1 cells expressing non-targeting shRNA (shNT), Rab35 shRNA (shRab35), or

shRab35 and Rab35 cDNA resistant to shRab35 (sh-res. Rab35). Dot plot shows the relative number of sEVs per cell. The number of sEVs in the sEV fraction was quantified using NanoSight. (**d**) Relative numbers of MCF-7 cells grown in the presence of CM compared to the number of cells grown in normal medium. CM was prepared from DXR-induced senescent RPE-1 cells expressing shNT, shRab35, or shRab35 and sh-res. Rab35. Statistical analysis was applied only to the data of culture day 3. Replicates are biological replicates ($n = 3$). Error bars indicate SD. *$p < 0.05$ [two-tailed *t*-test for (a) and one-way ANOVA with post-hoc Dunnett's two-tailed test for (c,d)].

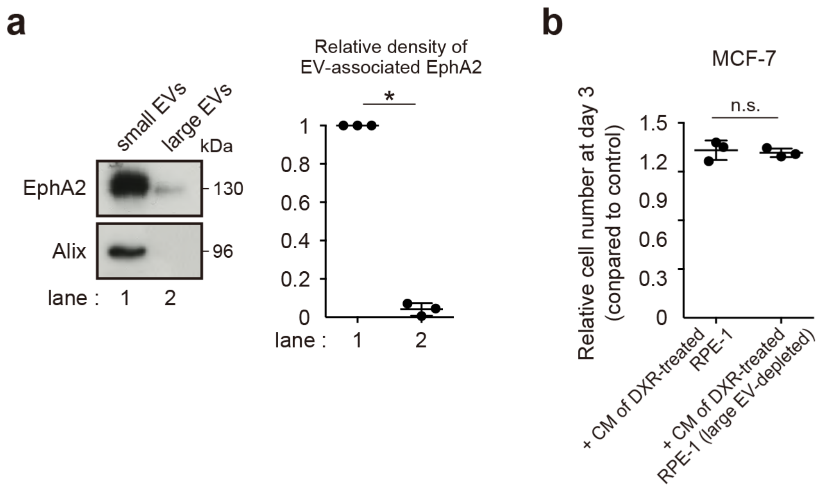

Supplementary Figure 2 | sEV is the major type of EphA2-containing EVs. (**a**) Immunoblotting of EphA2 and Alix in the small and large EV fraction prepared from DXR-induced senescent RPE-1 cells. Large EVs were prepared by pelleting them from the CM of DXR-induced senescent RPE-1 cells (10,000 × g, 30 min). Dot plot represents the relative density of EV-associated EphA2 analyzed by ImageJ. (**b**) Relative numbers of MCF-7 cells grown for 3 days in the presence of CM compared to the number of cells grown for 3 days in normal medium. CM was prepared from DXR-induced senescent RPE-1 cells and was used directly or after depleting large EVs by centrifugation (10,000 × g, 30 min). Replicates are biological replicates (*n* = 3). Error bars indicate SD. *p < 0.05 [two-tailed *t*-test].

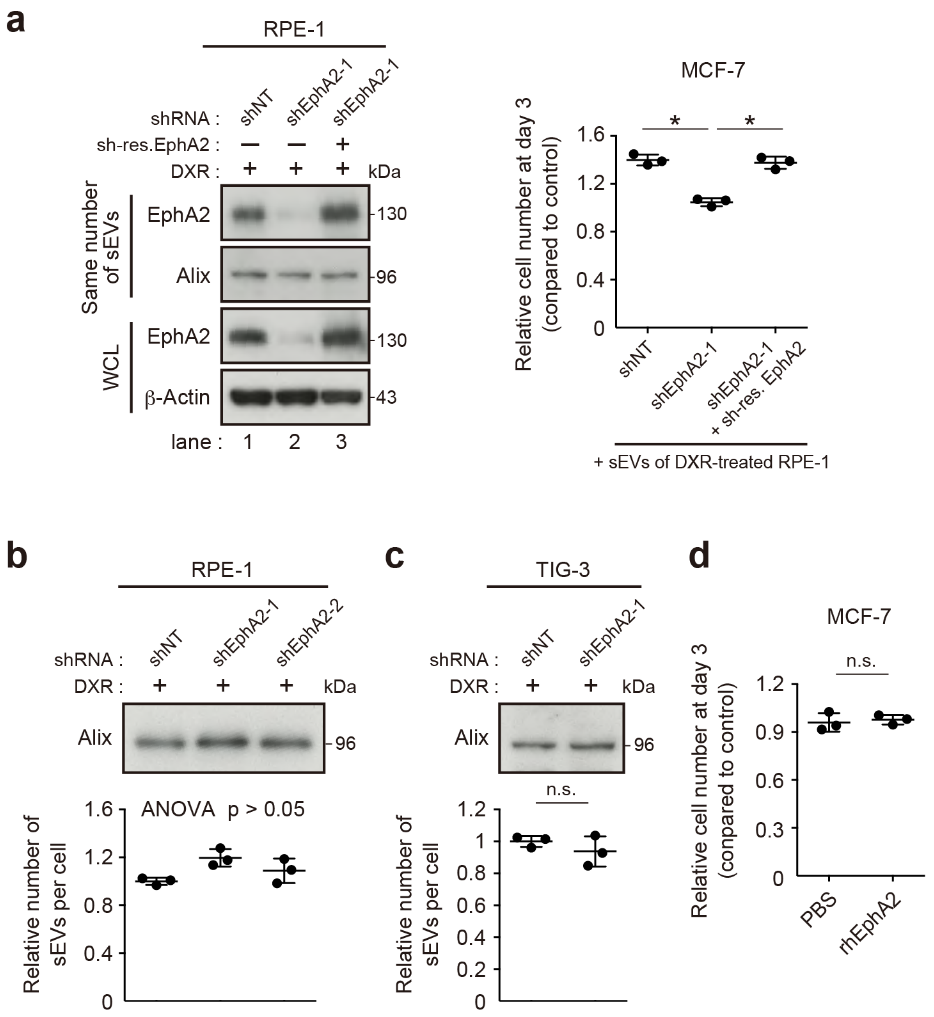

Supplementary Figure 3 | EphA2 is responsible for the pro-proliferative effect of sEVs. (**a**) Immunoblotting of EphA2 and Alix in the sEV fraction and of EphA2 and β-actin in the WCL of DXR-induced senescent RPE-1 cells expressing non-targeting shRNA (shNT), EphA2 shRNA (shEphA2-1), or shEphA2-1 and EphA2 cDNA resistant to shEphA2-1 (sh-res. EphA2). The numbers of sEVs were quantified in advance using NanoSight, and the same number of sEVs was loaded in each lane. Dot plot represents the relative numbers of MCF-7 cells grown for 3 days in the presence of sEVs compared to the numbers of cells grown for 3 days in normal medium. sEVs were purified from DXR-induced senescent RPE-1 cells expressing shNT, shEphA2-1, or shEphA2-1 and sh-res. EphA2. sEVs were added to the medium at a concentration of 2×10^9 particles/ml. (**b,c**) Dot plots show the relative number of sEVs per cell for DXR-induced senescent (**b**) RPE-1 and (**c**) TIG-3 cells expressing shNT or shEphA2 (shEphA2-1 or shEphA2-2). The number of sEVs in the sEV fraction was quantified using NanoSight. (**d**) Relative numbers of MCF-7 cells grown for 3 days in the presence of PBS or recombinant human EphA2 (1 μg/ml) in normal medium compared to the number of untreated cells grown for 3 days in normal medium. MCF-7 cells were plated at a density of 4×10^2 cells/cm^2 1 day before starting the experiments. Replicates are biological replicates ($n = 3$). Error bars indicate SD. *$p < 0.05$ [one-way ANOVA with post-hoc Dunnett's two-tailed test for (a,b) and two-tailed t-test for (c,d)].

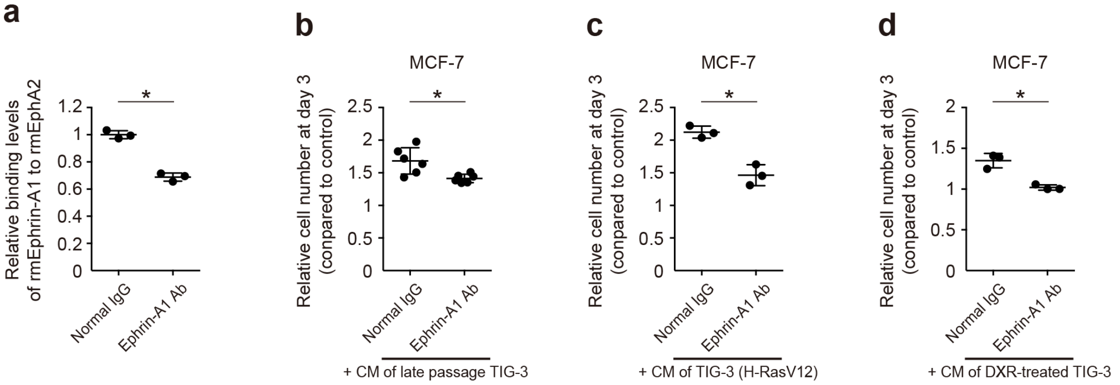

Supplementary Figure 4 | EphA2/ephrin-A1 signals are involved in the pro-proliferative effects of the CM of senescent TIG-3 cells. (**a**) Relative binding levels of mouse ephrin-A1-Fc to immobilized mouse EphA2-Fc in the presence of normal rabbit IgG or rabbit anti-ephrin-A1 IgG (100 µg/ml). (**b-d**) Relative numbers of MCF-7 cells grown for 3 days in the presence of CM compared to the number of cells grown for 3 days in normal medium. CM was prepared from (**b**) replicative senescent (late passage), (**c**) Ras-induced senescent, or (**d**) DXR-induced senescent TIG-3 cells. Normal rabbit IgG or anti-ephrin-A1 IgG was added to the CM at a concentration of 5 µg/ml. The densities of TIG-3 cells were as following at 1 day before starting CM preparation; 8×10^2 cells/cm^2 for replicative senescent TIG-3 cells; 1×10^4 cells/cm^2 for Ras-induced senescent TIG-3 cells; 5×10^3 cells/cm^2 for DXR-induced senescent TIG-3 cells. The experiments were technically replicated 3 times for (a) ($n = 3$) and biologically replicated 6 times for (b) ($n = 6$) and 3 times for (c,d) ($n = 3$). Error bars indicate SD. *$p < 0.05$ [two-tailed t-test].

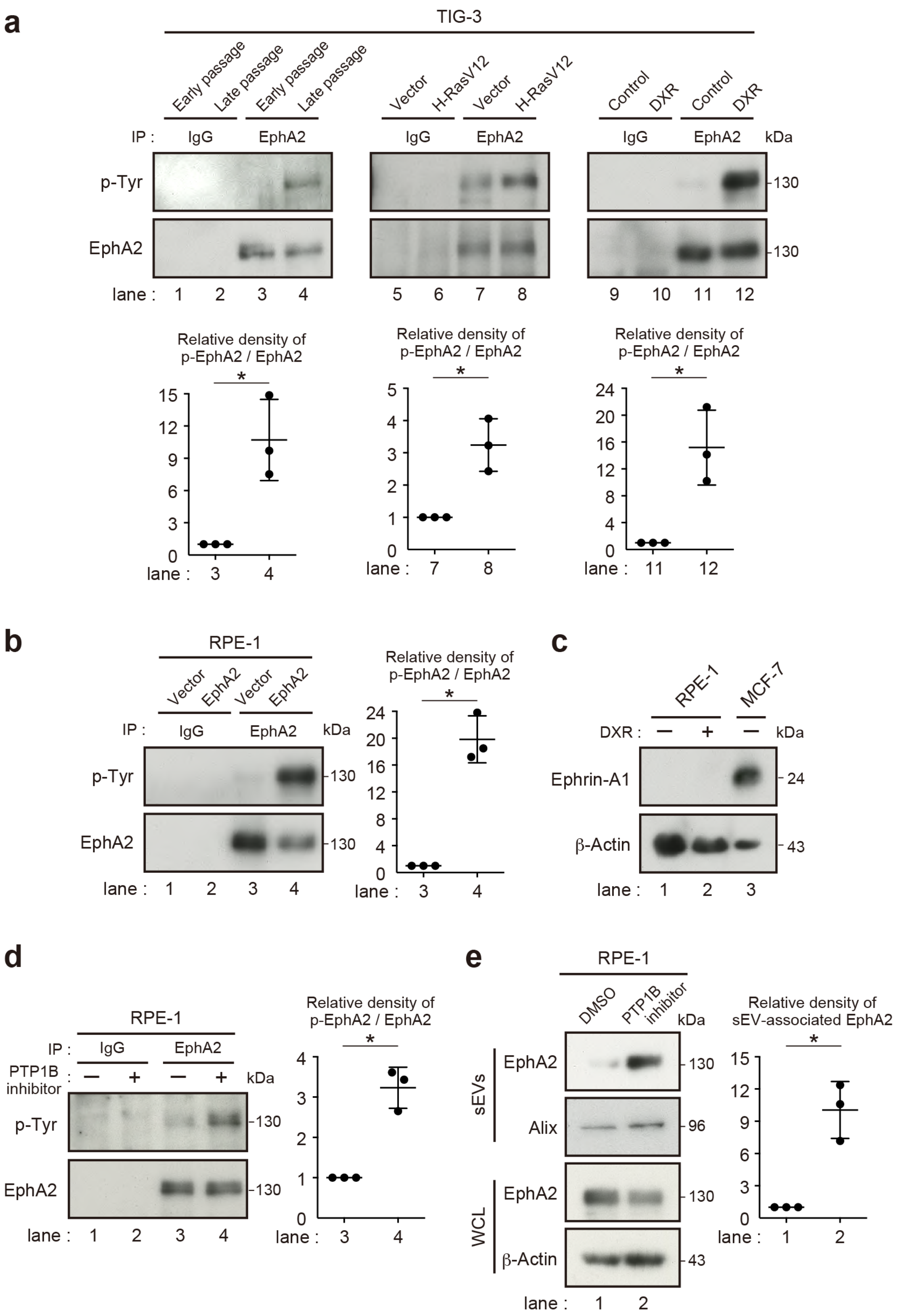

Supplementary Figure 5 | EphA2 phosphorylation is associated with its sorting into sEVs. (**a**) EphA2 immunoprecipitates prepared from pre-senescent control, replicative senescent (late passage), Ras-induced senescent, and DXR-induced senescent TIG-3 cells were immunoblotted with anti-phosphotyrosine and anti-EphA2 antibody. Dot plots represent the relative density of phospho-EphA2 / EphA2 analyzed by ImageJ. (**b**) EphA2 immunoprecipitates prepared from

pre-senescent RPE-1 cells expressing empty vector or ectopic EphA2. Dot plot represents the relative density of phospho-EphA2 / EphA2 analyzed by ImageJ. (**c**) Immunoblotting of ephrin-A1 and β-actin in the WCL of control and DXR-induced senescent RPE-1 cells and MCF-7 cells. (**d**) EphA2 immunoprecipitates prepared from pre-senescent RPE-1 cells treated for 3 days with DMSO or 20 μm PTP1B inhibitor (CAS:765317-72-4) were immunoblotted with anti-phosphotyrosine anti-EphA2 antibody. Dot plot represents the relative density of phospho-EphA2 / EphA2 analyzed by ImageJ. (**e**) Immunoblotting of EphA2 in the sEV fraction and WCL of pre-senescent RPE-1 cells treated for 3 days with DMSO or 20 μm PTP1B inhibitor. Dot plot represents the relative density of sEV-associated EphA2 analyzed by ImageJ. Replicates are biological replicates ($n = 3$). Error bars indicate SD. *$p < 0.05$ [two-tailed *t*-test].

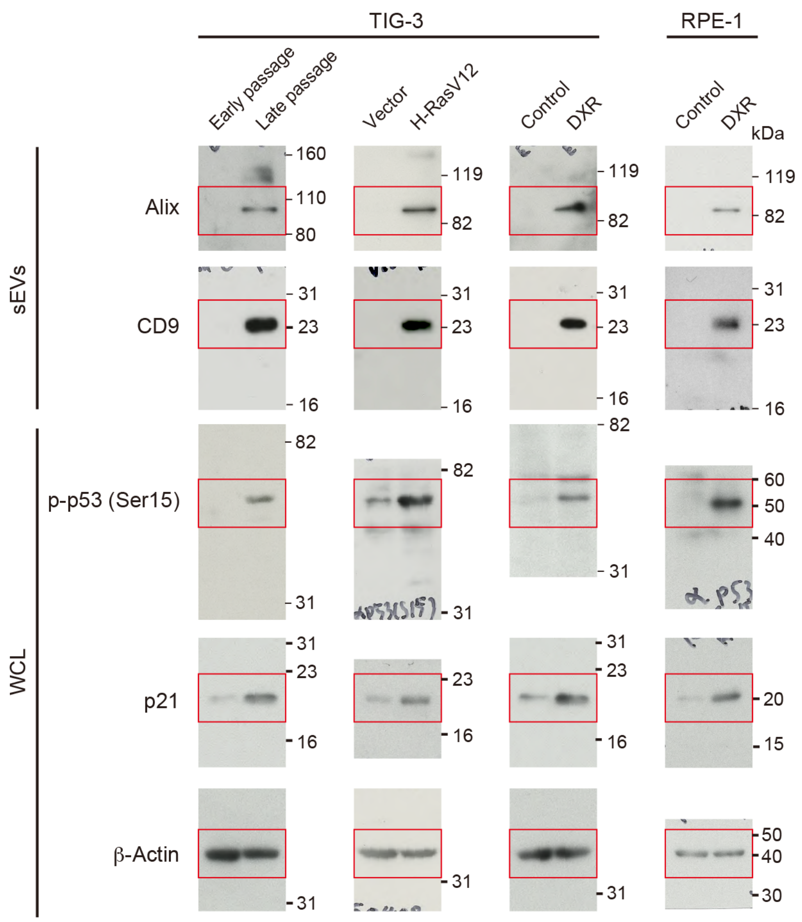

Supplementary Figure 6 | Uncropped gel images of western blots in Figure 1.

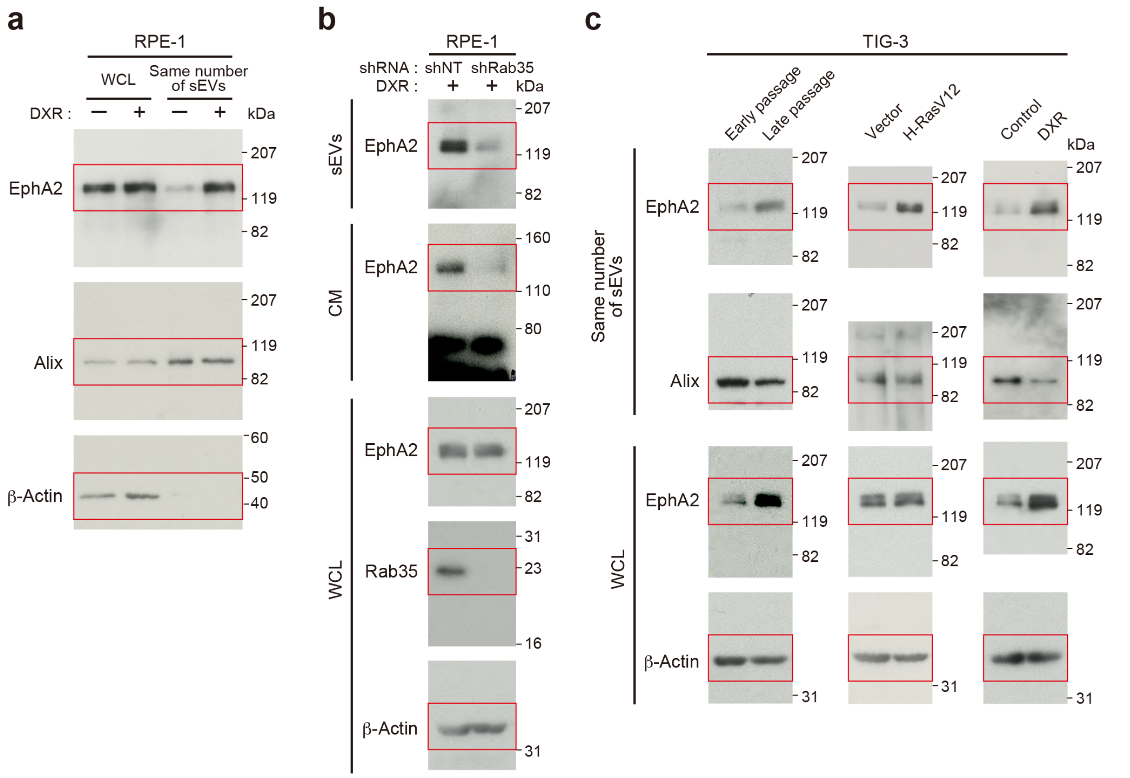

Supplementary Figure 7 | Uncropped gel images of western blots in Figure 3. (**a**) Figure 3b. (**b**) Figure 3c. (**c**) Figure 3d.

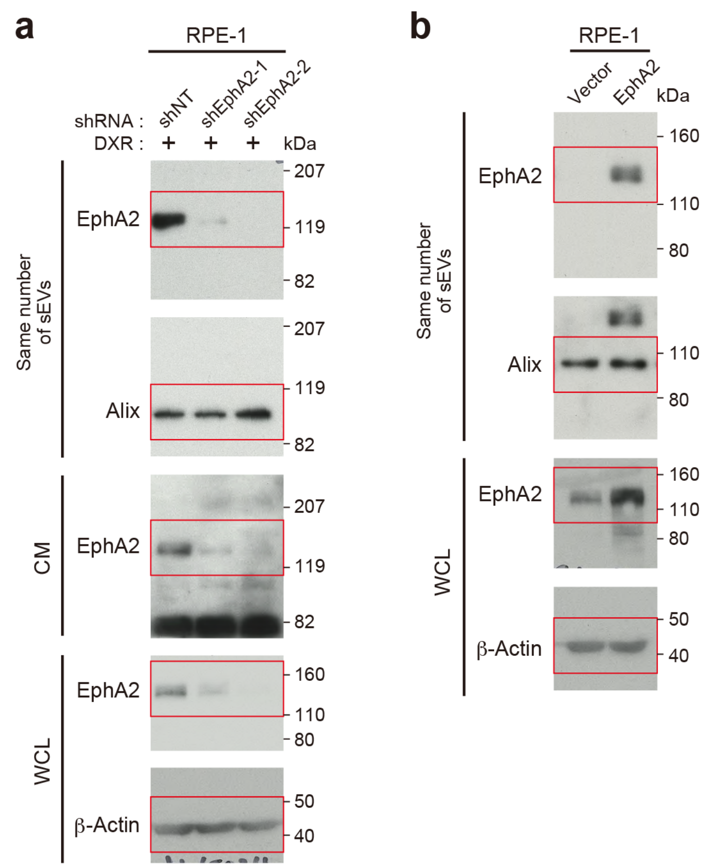

Supplementary Figure 8 | Uncropped gel images of western blots in Figure 4. (**a**) Figure 4a. (**b**) Figure 4b.

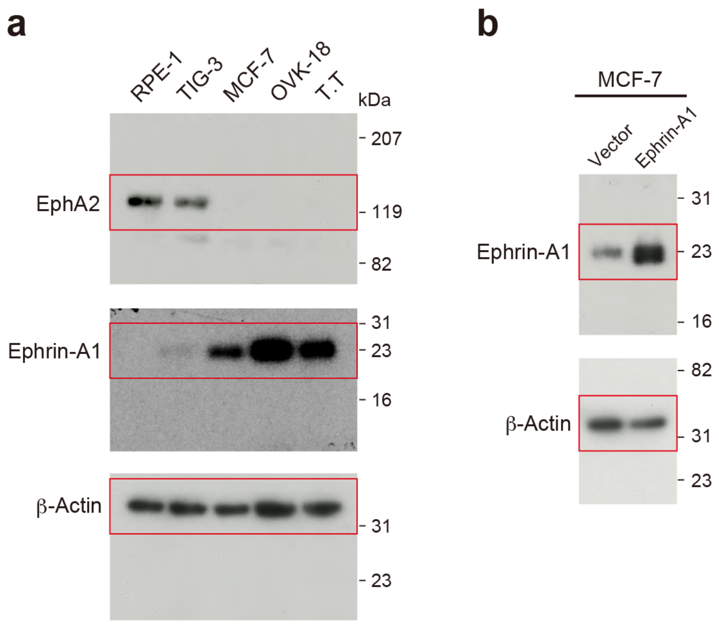

Supplementary Figure 9 | Uncropped gel images of western blots in Figure 5. (**a**) Figure 5a. (**b**) Figure 5c.

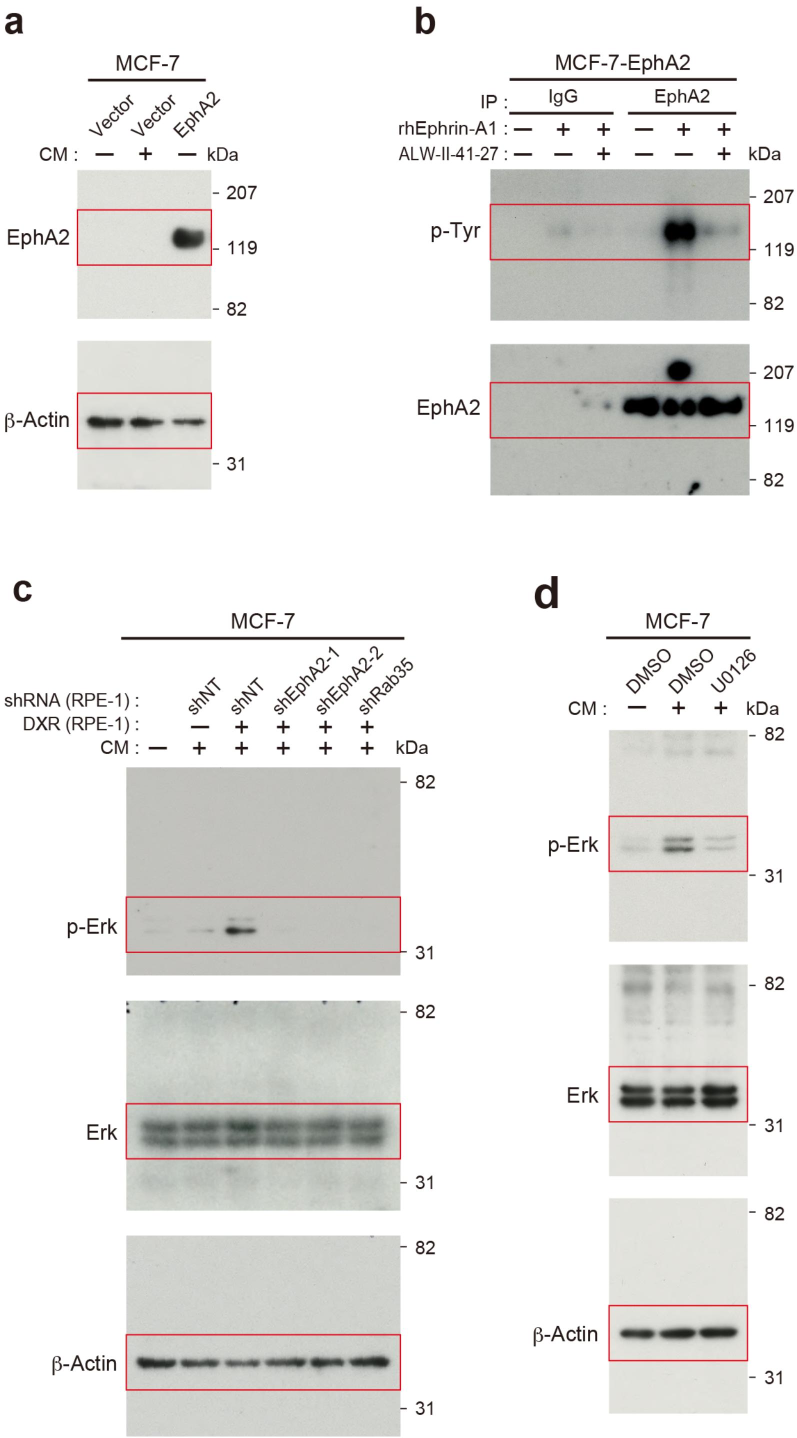

Supplementary Figure 10 | Uncropped gel images of western blots in Figure 6. (**a**) Figure 6a. (**b**) Figure 6b. (**c**) Figure 6d. (**d**) Figure 6g.

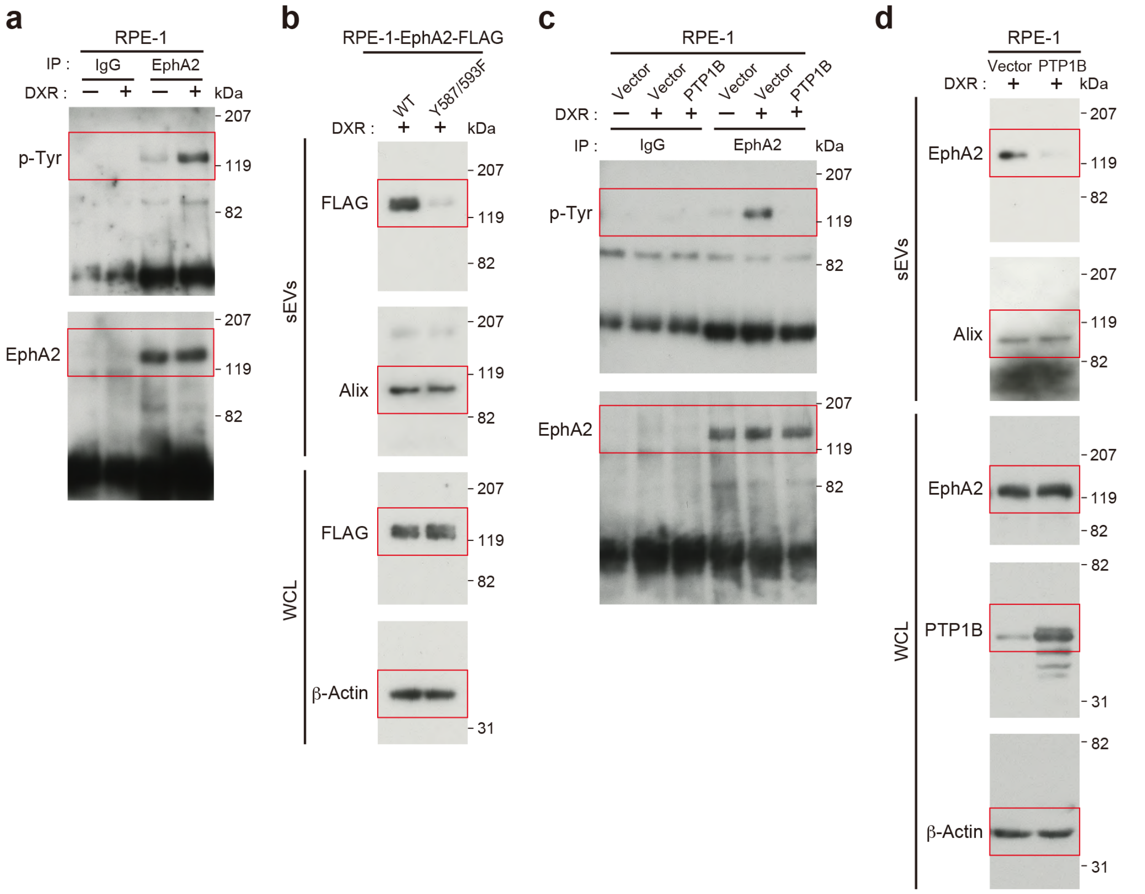

Supplementary Figure 11 | Uncropped gel images of western blots in Figure 7. (**a**) Figure 7a. (**b**) Figure 7b. (**c**) Figure 7e. (**d**) Figure 7f.

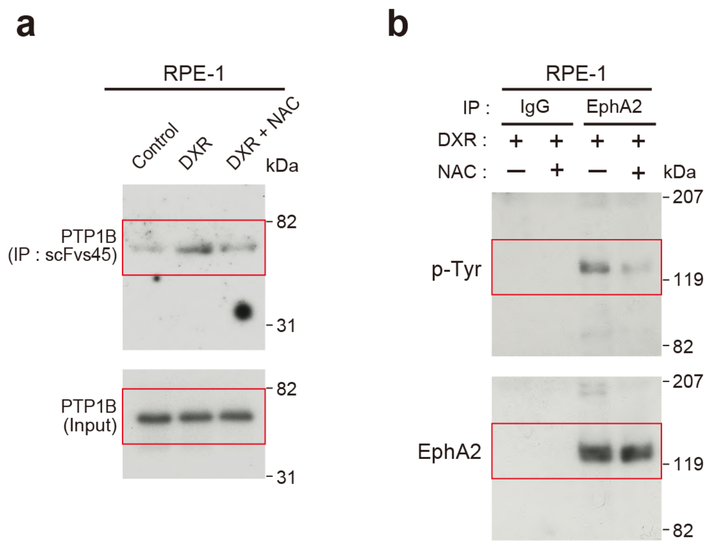

Supplementary Figure 12 | Uncropped gel images of western blots in Figure 8. (**a**) Figure 8b. (**b**) Figure 8d.
